# All‐trans retinoic acid and human salivary histatin‐1 promote the spreading and osteogenic activities of pre‐osteoblasts *in vitro*

**DOI:** 10.1002/2211-5463.12792

**Published:** 2020-02-11

**Authors:** Wei Sun, Andi Shi, Dandan Ma, Jan G. M. Bolscher, Kamran Nazmi, Enno C. I. Veerman, Floris J. Bikker, Haiyan Lin, Gang Wu

**Affiliations:** ^1^ The Affiliated Stomatology Hospital Zhejiang University School of Medicine Hangzhou China; ^2^ Key Laboratory of Oral Biomedical Research of Zhejiang Province Hangzhou China; ^3^ Department of Oral Biochemistry Academic Centre for Dentistry Amsterdam (ACTA) University of Amsterdam (UvA) and Vrije Universiteit Amsterdam (VU) The Netherlands; ^4^ Key Laboratory of Oral Medicine Guangzhou Institute of Oral Disease, Stomatology Hospital of Guangzhou Medical University Guangzhou Medical University China; ^5^ Savaid Stomatology School Hangzhou Medical College China; ^6^ Department of Oral Implantology and Prosthetic Dentistry Academic Centre for Dentistry Amsterdam (ACTA) University of Amsterdam (UvA) and Vrije Universiteit Amsterdam (VU) The Netherlands

**Keywords:** all‐trans retinoic acid, cell spreading, histatin‐1, osteogenic cells, pre‐osteoblasts

## Abstract

Cell‐based bone tissue engineering techniques utilize both osteogenic cells and biomedical materials, and have emerged as a promising approach for large‐volume bone repair. The success of such techniques is highly dependent on cell adhesion, spreading, and osteogenic activities. In this study, we investigated the effect of co‐administration of all‐trans retinoic acid (ATRA) and human salivary peptide histatin‐1 (Hst1) on the spreading and osteogenic activities of pre‐osteoblasts on bio‐inert glass surfaces. Pre‐osteoblasts (MC3T3‐E1 cell line) were seeded onto bio‐inert glass slides in the presence and absence of ATRA and Hst1. Cell spreading was scored by measuring surface areas of cellular filopodia and lamellipodia using a point‐counting method. The distribution of fluorogenic Hst1 within osteogenic cells was also analyzed. Furthermore, specific inhibitors of retinoic acid receptors α, β, and γ, such as ER‐50891, LE‐135, and MM‐11253, were added to identify the involvement of these receptors. Cell metabolic activity, DNA content, and alkaline phosphatase (ALP) activity were assessed to monitor their effects on osteogenic activities. Short‐term (2 h) co‐administration of 10 μm ATRA and Hst1 to pre‐osteoblasts resulted in significantly higher spreading of pre‐osteoblasts compared to ATRA or Hst1 alone. ER‐50891 and LE‐135 both nullified these effects of ATRA. Co‐administration of ATRA and Hst1 was associated with significantly higher metabolic activity, DNA content, and ALP activity than either ATRA or Hst1 alone. In conclusion, co‐administration of Hst1 with ATRA additively stimulated the spreading and osteogenicity of pre‐osteoblasts on bio‐inert glass surfaces *in vitro*.

## Graphical abstract

Adhesion and spreading of osteogenic cells are indispensable for initiation of bone regeneration. We showed that the co‐administration of all‐trans retinoic acid (ATRA) and human salary histatin‐1 (Hst1) resulted in significantly enhanced spreading, metabolic activity, proliferation (DNA content), and osteogenic differentiation (alkaline phosphatase activity) of pre‐osteoblasts on bio‐inert surfaces, thus bearing a promising application potential in bone tissue engineering.

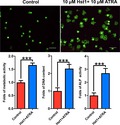

**Abbreviations**
ALP: alkaline phosphatase
ATRA: all‐trans retinoic acid
FAK: focal adhesion kinase
Fmoc: fluorenylmethoxycarbonyl
Hst1: histatin‐1
LVBD: large‐volume bone defects
pNPP: *p*‐nitrophenyl phosphate
RXR: retinoid X receptor

Large‐volume bone defects (LVBD) may severely influence aesthetics and musculoskeletal functions. Due to the limited healing capacity of bone tissues, the osseous repair of LVBD can be problematic 1. For treatment purposes, autologous bone grafts are still considered as the gold standard. However, their application is confined by limited graft supply, donor site pain and morbidity, infections, and poor cosmetic outcomes 2. As alternative options to autologous bone grafts, allografts, xenografts, and synthetic materials have been developed and adopted as bone‐defect‐filling materials 3. However, most of these materials need to be premixed with autologous bone grafts to obtain osteogenic cells. In such cases, the disadvantages of autologous bone grafts remain.

To approach these challenges, cell‐based tissue engineering techniques that integrate osteogenic cells and biomedical materials have emerged as a promising approach for bone repair 4. However, the chance of success is, however, highly dependent on the interactions of the osteogenic cells with the cell‐scaffold surfaces 5. At first, cell–substrate interactions are critical for the determination of cell fates, such as proliferation, quiescence, or apoptosis 6. Furthermore, surface adhesion and osteogenic cell proliferation are indispensable for initiation of bone regeneration 7. Consequently, tremendous efforts have been made to develop a large variety of techniques (e.g., immobilized RGD peptide on titanium surface 8 and femtosecond laser‐induced micropattern and Ca/P deposition 9) to modify surface chemistry and/or topography of various biomedical materials in order to improve their cell–substrate interactions 5, 10. These material‐specific approaches, however, render their broad applicability limited. In comparison, the other strategies – cell‐targeting techniques that directly promote cellular response to materials – have become highly attractive as they do not require surface modifications of materials, thus bearing a broader applicability.

A promising candidate cell‐targeting agent to promote cell–substrate interactions is histatin‐1 (Hst1), a member of a large histidine‐rich salivary peptide family. Our previous findings show that Hst1 can significantly promote the attachment, spreading, and migration of various cell types including epithelial, endothelial, and osteogenic cells 11, 12, 13, 14, 15. Our recent data confirm that Hst1 can promote the spreading of osteogenic cells on both bio‐inert glass and titanium surface 14, 15, 16, 17, which suggests a promising application potential of Hst1 in the cell‐based bone tissue engineering.

In a previous study, we found that a 3‐day treatment of all‐trans retinoic acid (ATRA), an active metabolite of vitamin A, can cause the uniform alignment and stretch of cell skeleton (Fig. S1). This finding inspired us to apply ATRA to promote cell spreading. ATRA, the active metabolite of vitamin A, is known to act as regulator of many physiologic processes 18. It plays a role in a wide range of biological processes mediated through binding and activation of the nuclear receptors, such as the RA receptor (RAR) and retinoid X receptor (RXR). There are three subtypes of RAR (α, β, and γ) and three subtypes of RXR (α, β, and γ). RARs are bound and activated by ATRA, while RXRs are bound and activated by the 9‐cis‐RA only 19. Heterodimers of activated RAR and RXR act as ligand‐dependent transcription factors. On the other hand, it was found that a 3‐day treatment of ATRA also results in significantly reduced osteogenic differentiation of pre‐osteoblast cells and bone marrow stromal cells 20, 21. Consequently, in the present study, we analyzed *in vitro* the effect of a short (2 h) co‐application of ATRA and Hst1 in order to amplify the stimulating effect of Hst1 on the spreading of osteogenic cells on the one hand and to avoid the decrease in osteogenic potential on the other hand.

## Materials and methods

### Study design

The effect of a short (2 h) co‐administration of ATRA and Hst1 on cell spreading was evaluated. Thereafter, we used specific inhibitors of retinoic acid receptor alpha (RARα), RARβ, and RARγ, that is, ER‐50891, LE‐135, and MM‐11253, respectively, to identify the involvement of RARs. Furthermore, we examined the effects of a short co‐administration of ATRA and Hst1 on the osteogenic potentials of pre‐osteoblast cells, such as metabolic activity, DNA content (indicator for proliferation), and alkaline phosphatase (ALP) activity (early marker of osteogenic differentiation).

### Preparation of histatin‐1

Histatin‐1 was manufactured by solid‐phase peptide synthesis using 9‐fluorenylmethoxycarbonyl (Fmoc) chemistry as described previously 15, 22. Hst1 was purified to at least 95% by high‐performance liquid chromatography (RF‐HPLC, Dionex Ultimate 3000; Thermo Scientific, Breda, the Netherlands). The authenticity was confirmed by mass spectrometry with a Microflex LRF MALDI‐TOF (Bruker Daltonik GmbH, Bremen, Germany) as previously described 15, 22. Fluorescently labeled Hst1 was prepared using the fluorogenic dye ATTO‐647N (ATTO‐TEC GmbH, Siegen, Germany). The ε‐amino group of the side chain of lysine residue number 17 (lys17, K of Hst1 after removal of the specific protective lysine derivative, Fmoc‐Lys(ivDde)‐OH, by hydrazine (2% hydrazine hydrate)) was coupled to equimolar amount of the dye.

### Cell culture and chemicals

MC3T3‐E1, a mouse pre‐osteoblast cell line, subclone 4 (CRL‐2593, American Type Culture Collection, ATCC, Manassas, VA, USA), was cultured in alpha‐minimum essential medium (α‐MEM; Gibco, Thermo Fisher Scientific, Paisley, UK) supplemented with 10% FBS (Gibco, Thermo Fisher Scientific) and 1% penicillin/streptomycin (Sigma, St. Louis, MO, USA). Cells were cultured in humidified oxygen‐controlled 37 °C incubator with 5% CO_2_. Passages between 4 and 7 were used for experiments.

### Measurement of cell spreading on glass surface

Cells were treated with serum‐free medium for 24 h before being detached by 0.05% trypsin (Gibco, Thermo Fisher Scientific). Growth medium contained 2% FBS was used to inactivate the effect of trypsin and to resuspend the cells. MC3T3‐E1 was seeded on coverslips (20 mm in diameter; Thermo Scientific, Braunschweig, Germany) in 12‐well plates at a density of 6 × 10^4^ cells/well. Cells were treated either with 0, 1, 10, or 20 µm ATRA (Sigma‐Aldrich) or Hst1 or co‐administered 10 µm ATRA and Hst1. To investigate the role of potential signaling pathways, 10 µm RARα antagonist (ER‐50891; R&D, Bio‐Techne, Minneapolis,  MN, USA), 10 µm RARβ antagonist (LE‐135; R&D, Bio‐Techne), and 10 µm RARγ antagonists (MM‐11253; R&D, Bio‐Techne) were supplemented in cell spreading assays. Cells were photographed every 20 min for 3 h using a microscope (EVOS FL; Thermo Fisher Scientific) equipped with a LPlanFL PH2 20× using the phase‐contrast setting or the Cy5 light cube (628/40 and 692/40 nm, excitation and emission filters, respectively). Relative cell spreading surface area was quantified by measuring the surface area of cells' filopodia and lamellipodia using a manual point‐counting method 23 (Fig. S2). Each assay was performed in triplicate and repeated twice.

### Fluorescent staining of spreading cells

Cell spreading on glass surface was performed as described in the section of cell spreading assay. 1.5 h after seeding, cells were fixed, dehydrated, and stained with FITC‐Phalloidin. Fluorescent micrographs were randomly taken using a fluorescent microscope (Leica Microsystems GmbH, Wetzlar, Germany) with excitation/emission wavelengths (nm) of 496/516. On the micrographs, spreading surface of each cell was estimated using the above‐mentioned point‐counting method. More than 20 cells per group were calculated.

### Cell metabolic activity

Subconfluent growing cells were plated on glass coverslips (diameter, 10 mm; Thermo Scientific, Germany) in 48‐well plate in a density of 1.5 × 10^4^ cells/well. Cells were treated with either 10 µm Hst1 or ATRA, or cells were treated with premixed ATRA and Hst1 for 2 h at 37 °C. After washing with 1× PBS, cells were treated with α‐MEM with 10% FBS which was refreshed on a daily basis. PrestoBlue™ Cell Viability Assay was adopted to evaluate cell viability using the reducing ability of cells (Invitrogen Corporation, Carlsbad, CA, USA). In short, 1/10th volume of PrestoBlue™ reagent was added to cells in culture medium and incubated for 30 min at 37 °C. Results were measured by reading fluorescence intensity with the Multiskan FC (Thermo Scientific) using a fluorescence excitation wavelength of 560 nm and an emission wavelength of 590 nm. Each assay was performed in triplicate and repeated twice.

### DNA quantification

The CyQUANT Proliferation Assay Kit (Molecular Probes, Waltham, MA, USA) was employed to monitor the proliferation of pre‐osteoblasts. Subconfluent growing cells were plated on glass coverslips (diameter, 10 mm; Thermo Scientific, Germany) in a 48‐well plate at a density of 1.5 × 10^4^ cells/well. Cells were treated with either 10 µm Hst1 or ATRA, or co‐administered ATRA and Hst1 for 2 h. After washing with 1× PBS, cells were treated with α‐MEM with 10% FBS which was changed every day. The cells were retrieved right after or 5 days after the short treatment. Subsequently, the freshly prepared 100 μL CyQUANT solution was added to the well to measure the optical density with excitation at 480 nm and emission at 520 nm using a plate reader (Synergy, BioTek™, Winooski, VT, USA). Each assay was performed in triplicate and repeated twice.

### Alkaline phosphatase assays

Quantitative determination of ALP activity was done using the *p*‐nitrophenyl phosphate (pNPP) liquid substrate method. Cells were suspended in serum‐free media in the presence or absence of 10 µm Hst1 or 10 µm ATRA or both and then seeded on glass coverslips (10 mm in diameter; Thermo Scientific, Germany) in 48‐well plates at a density of 5 × 10^4^ cells/well. Two hours after seeding, the media were changed to 10% FBS‐containing α‐MEM, and subsequently, cells were cultured for 1 more day. Thereafter, cells were treated with α‐MEM containing 2% FBS. After 3 days, cells were lysed in distilled water using a freeze–thaw method and harvested with a cell scraper. Cell lysates were centrifuged at 250 ***g*** for 5 min at room temperature, and supernatants were incubated with 1.86 mg·mL^−1^ pNPP for 1 h at 37 °C in the dark. After 1 h, 100 μL 300 mm NaOH solution was added; then, absorbance at 405 nm was measured using Multiskan FC (Thermo Scientific, Rockford, IL, USA), and ALP activity was calculated according to the standard curve. The total protein was assessed using Pierce BCA Protein Assay Kit (Thermo Fisher Scientific, Rockford, IL, USA) for normalizing the ALP activity 20. Each assay was performed in quadruplicate and repeated twice.

### Statistical analysis

Data were plotted using graphpad prism (GraphPad Software version 6.0, La Jolla, CA, USA) and analyzed by one‐way ANOVA with Bonferroni's *post hoc* test for multiple comparisons. For the data from different groups at different time points in Fig. 2C, we used two‐way ANOVA to analyze the data with Tukey test for multiple comparisons. Results were reported as mean ± standard deviation (SD). A value of *P* < 0.05 was considered as statistical significance.

## Results and Discussion

### ATRA and Hst1 promote the spreading of osteogenic cells on bio‐inert glass surface subsection

**Figure 1:**
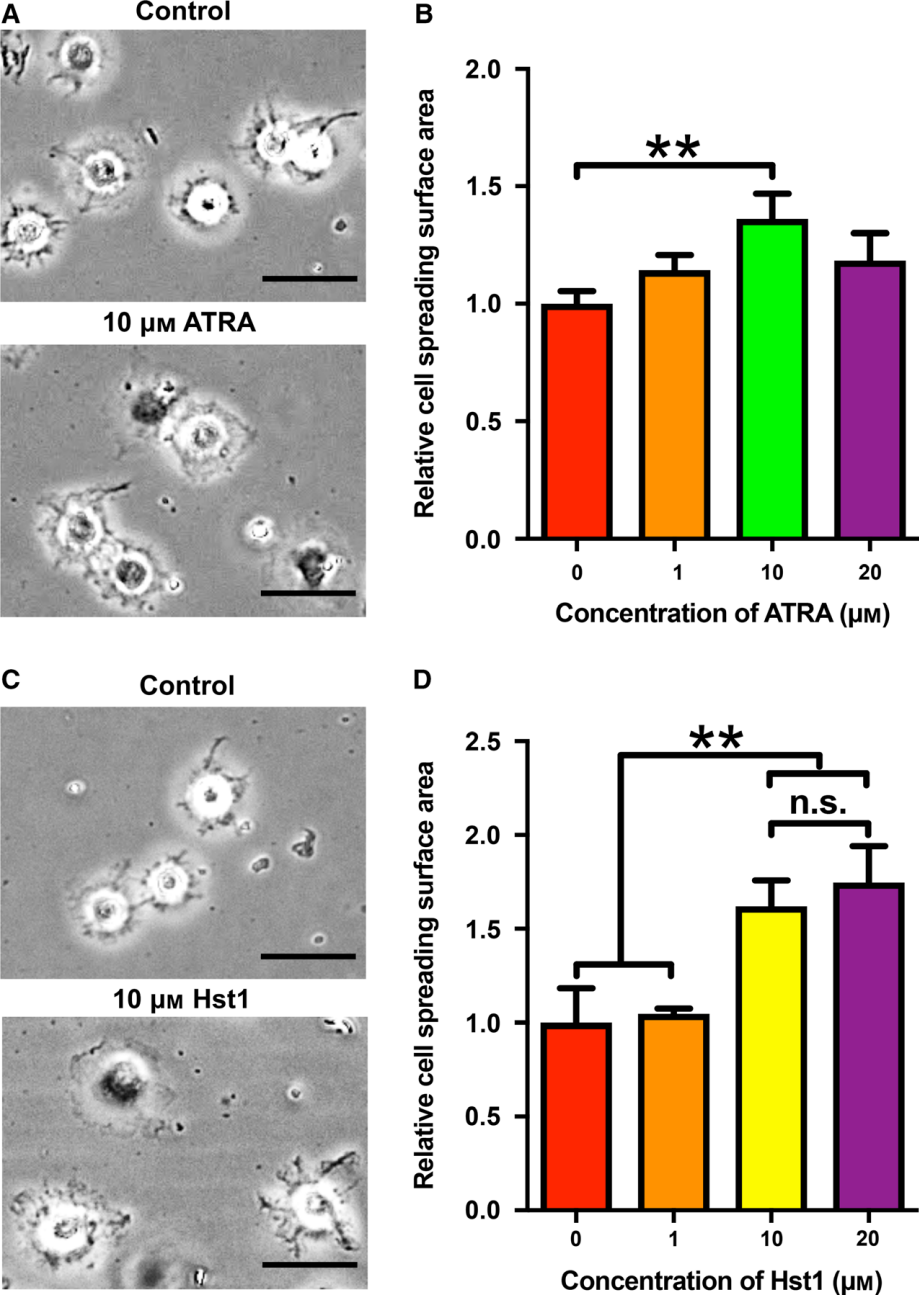
(A) Light micrographs depicting the spreading of pre‐osteoblasts in the presence or absence of 10 µm ATRA. Bar = 50μm. (B) Folds of cell spreading surface area in the presence or absence of 1, 10, and 20 µm ATRA. Data are shown as mean ± SD (*n* = 6). (C) Light micrographs of the cell spreading in the presence or absence of 10 µm Hst1. Bar = 50μm. (D) Folds of cell spreading surface area in the presence or absence of 1, 10, and 20 µm Hst1. Data were shown as mean ± SD (*n* = 6). Data were plotted using graphpad prism (GraphPad Software version 6.0) and analyzed by one‐way ANOVA with Bonferroni's *post hoc* test for multiple comparisons. **P < 0.01 comparing with control group; n.s., no statistically significant difference.

At a concentration of 10 µm, ATRA significantly promoted the spreading of pre‐osteoblasts on bio‐inert glass surfaces in comparison with the control (no ATRA) (Fig. 1A,B). Hst1 at 10 and 20 µm significantly promoted the spreading of osteogenic cells in comparison with the control (no Hst1) (Fig. 1C,D). Thereafter, we performed a pilot experiment to check the effect of co‐administered 10 µm Hst1 and ATRA of different concentrations (e.g., 0.1, 1, and 10 µm). Only 10 µm ATRA and 10 µm Hst1 resulted in a significant cell spreading area than 10 µm Hst1 effect (data not shown). Therefore, we adopted the combination of 10 µm ATRA and 10 µm Hst1. Our data showed that there was no significant difference between the promoting effects of 10 µm Hst1 and 20 µm Hst1. In this light, it was chosen to further use 10 µm ATRA and 10 µm Hst1 in the following experiments.

### Effects of ATRA and Hst1 co‐administration on spreading of pre‐osteoblasts

**Figure 2:**
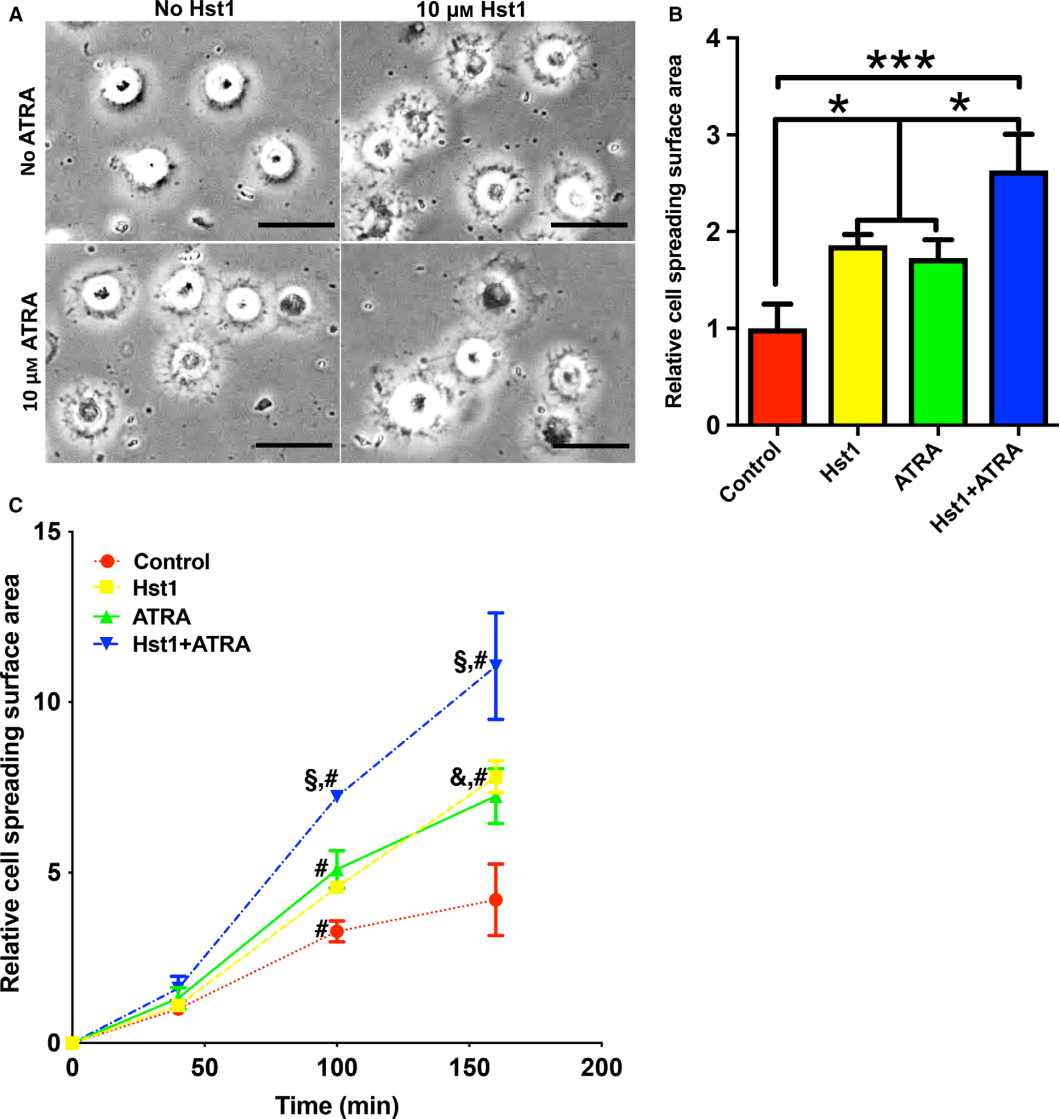
(A) Light micrographs depicting the spreading of pre‐osteoblasts in the presence or absence of 10 µm Hst1 or 10 µm ATRA. Bar = 50μm. (B) Folds of spreading surface area of pre‐osteoblasts in the presence or absence of 10 µm Hst1 or 10 µm ATRA. Data are shown as mean ± SD (*n* = 6). Data were plotted using graphpad prism (GraphPad Software version 6.0) and analyzed by one‐way ANOVA with Bonferroni's *post hoc* test for multiple comparisons. *P < 0.05; ***P < 0.001. (C) Time‐dependent cell spreading surface area [expressed in folds with the value of the control group (no Hst1 and no ATRA) at first time point as 1] in the presence or absence of 10 µm Hst1 or 10 µm ATRA. Data were shown as mean ± SD (*n* = 6). Data were analyzed by two‐way ANOVA with Tukey test for multiple comparisons. ^§^ *P* < 0.05 indicating a significant difference compared with the values in the groups of Hst1 or ATRA; ^&^ *P* < 0.05 indicating a significant difference compared with the value in the control group at the same time point; ^#^ *P* < 0.05 indicating a significant difference compared with the value in the same treatment group at the earlier time point.

**Figure 3:**
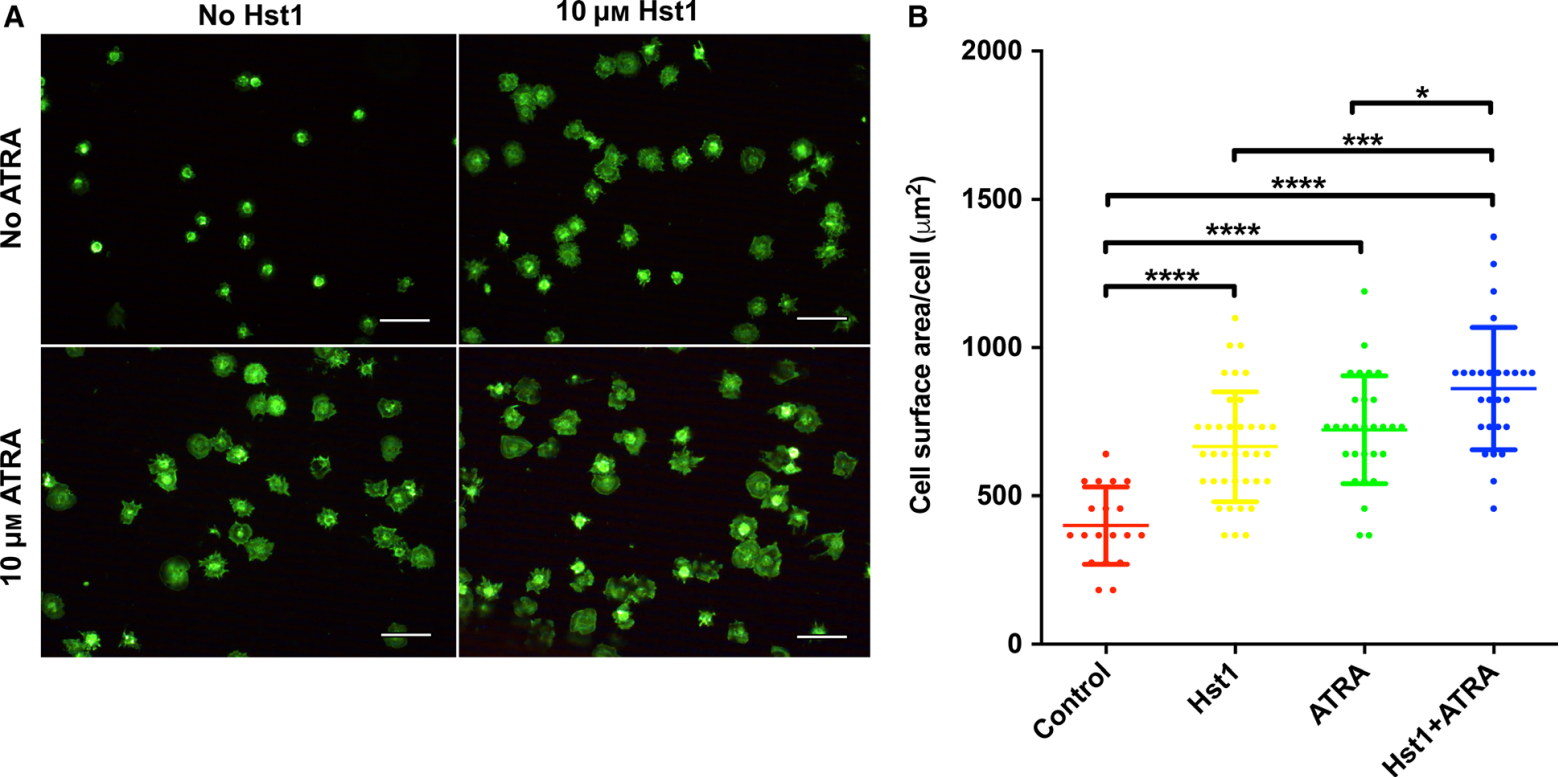
(A) Fluorescent micrographs depicting the spreading of pre‐osteoblasts (stained with FITC‐Phalloidin) in the presence or absence of 10 µm Hst1 and 10 µm ATRA on bio‐inert glass surface. Bar = 50μm. (B) The surface area per pre‐osteoblast in the presence or absence of 10 µm Hst1 and 10 µm ATRA on bio‐inert glass surface. Data were plotted using graphpad prism (GraphPad Software version 6.0) and analyzed by one‐way ANOVA with Bonferroni's *post hoc* test for multiple comparisons. Data were shown as mean ± SD (*n* > 20). *P < 0.05; ***P < 0.001; ****P < 0.0001.

Sixty minutes post‐treatment, the co‐administration of ATRA and Hst1 resulted in significantly larger spreading of pre‐osteoblasts (2.6‐fold) compared to the individual counterparts, viz. Hst1 (1.9‐fold) and ATRA (1.7‐fold) alone (Fig. 2A,B). In the subsequent time‐course assay, the promoting effect of 10 µm ATRA and 10 µm Hst1 became significant from 40 to 100 min and more pronounced at 160 min (Fig. 2C). Moreover, the surface area of cells treated with ATRA and Hst1 (861 ± 206 μm^2^) was also significantly higher than those treated with ATRA (723 ± 182 μm^2^, *P* < 0.05) or Hst1 alone (665 ± 185 μm^2^, *P* < 0.001) (Fig. 3A,B).

### The antagonists of RARα and RARβ suppressed the promoting effect of ATRA and Hst1 on cell spreading

**Figure 4:**
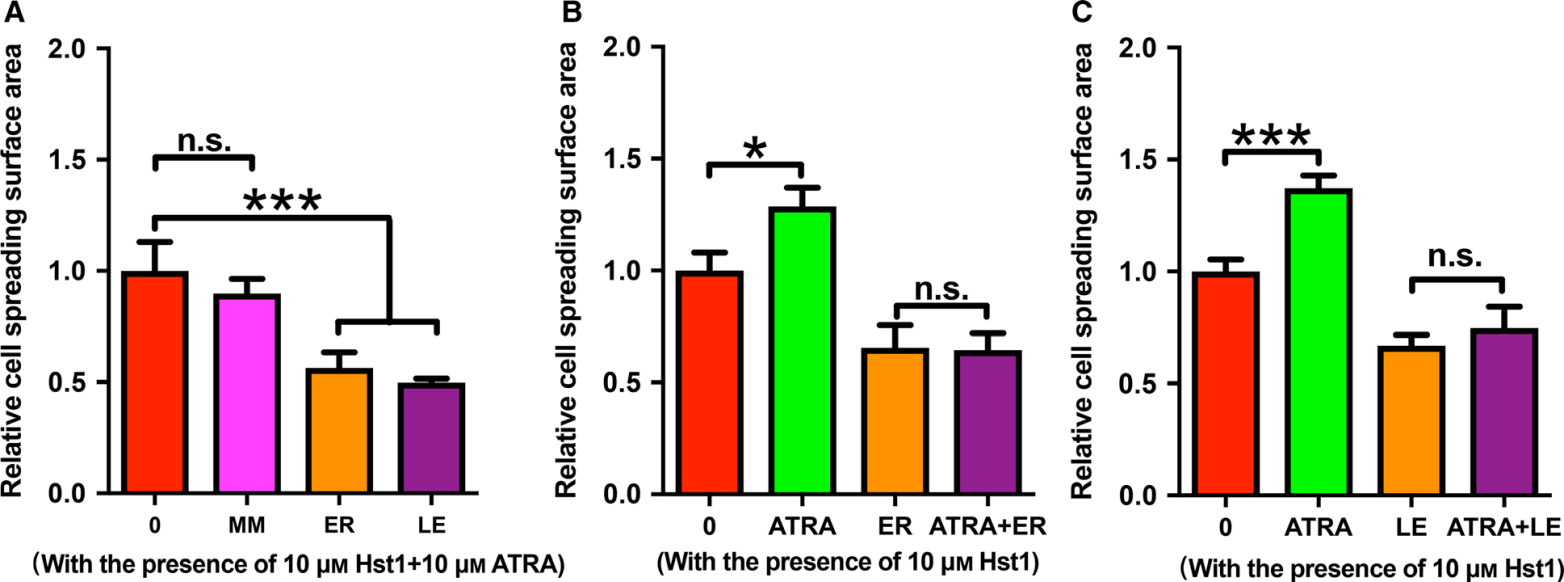
Folds of spreading surface area of pre‐osteoblasts that were treated with (A) co‐administered 10 µm ATRA and Hst1 with or without the pretreatment with MM [10 µm MM‐11253, the antagonist of retinoic acid receptor (RAR)γ], ER (10 µm ER‐50891, the antagonist of RARα), and LE (10 µm LE‐135, the antagonist of RARβ); (B) 10 µm Hst1 in the presence or absence of 10 µm MM or 10 µm ATRA; (C) co‐administered 10 µm Hst1 in the presence or absence of 10 µm LE135 or 10 µm ATRA. Data were plotted using graphpad prism (GraphPad Software version 6.0) and analyzed by one‐way ANOVA with Bonferroni's *post hoc* test for multiple comparisons. Data were shown as mean ± SD (*n* = 6). *P < 0.05; ***P < 0.001.

The antagonists of RARα (ER‐50891) and RARβ (LE‐135) significantly suppressed the promoting effect of the co‐administered ATRA and Hst1 on the spreading of pre‐osteoblasts (Fig. 4A). Consistent with above‐mentioned results, 10 µm ATRA significantly elevated the promoting effects of 10 µm Hst1 (*P* < 0.05), which could be nullified by the pretreatment of 10 µm ER‐50891 (Fig. 4B) or 10 µm LE‐135 (Fig. 4C).

### Co‐administration of ATRA and Hst1 upregulated the osteogenic activities of pre‐osteoblasts

**Figure 5:**
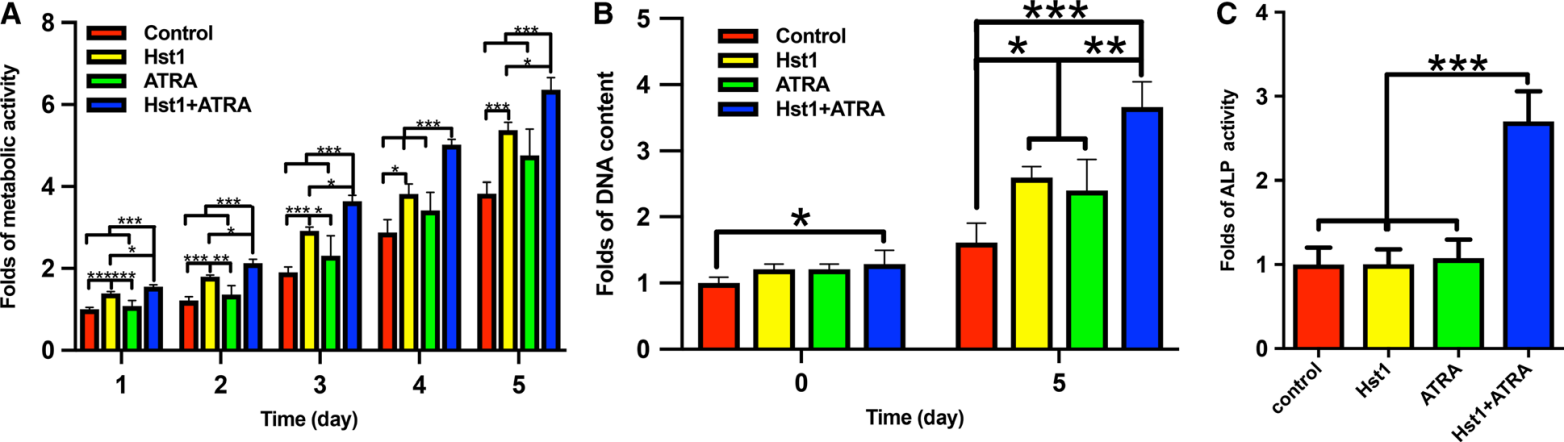
(A) Folds of the metabolic activities of pre‐osteoblasts within 5 days after a short (2 h) treatment with either no Hst1, no ATRA (control), or 10 µm Hst1, or 10 µm ATRA, or co‐administered 10 µm ATRA and 10 µm Hst1 with α‐MEM containing 10% FBS during seeding (*n* = 6). (B) Folds of DNA content at 0 day and 5 days after response graph of DNA content after a short (2 h) treatment with either no Hst1, no ATRA (control), or 10 µm Hst1, or 10 µm ATRA, or co‐administered 10 µm ATRA and 10 µm Hst1 with α‐MEM containing 10% FBS during seeding (*n* = 6). (C) Folds of ALP activity at 3 days after a short (2 h) treatment either without Hst1 or ATRA (control), or with 10 µm Hst1, or 10 µm ATRA, or co‐administered 10 µm ATRA and 10 µm Hst1 with α‐MEM containing 2% FBS during seeding (*n* = 8). Data were plotted using graphpad prism (GraphPad Software version 6.0) analyzed by one‐way ANOVA with Bonferroni's *post hoc* test for multiple comparisons. Data were shown as mean ± SD. *P < 0.05; **P < 0.01; ***P < 0.001.

Two‐hour treatment of 10 μm Hst1 significantly enhanced the metabolic activity of pre‐osteoblasts already after 1 day, in contrast to ATRA (Fig. 5A). Furthermore, the co‐administration of ATRA and Hst1 resulted in significantly higher metabolic activity in comparison with Hst1 alone (*P* < 0.05) (Fig. 5A). Directly after seeding, the DNA content in cells treated with Hst1 and ATRA was significantly higher than those stimulated by Hst1 or ATRA alone. Five days after seeding, the DNA content in the group of ATRA alone and Hst1 alone was significantly higher compared to the control group. The co‐administration of ATRA and Hst1 resulted in a significantly higher DNA content than those in the groups of ATRA or Hst1 alone (Fig. 5B). Three days postseeding, the ALP activity of the cells treated with Hst1 and ATRA was about 2.6‐fold higher (*P* < 0.001) than those in the groups of Hst1 alone, ATRA alone, or control (Fig. 5C).

Surface adhesion, spreading, proliferation, and differentiation of osteogenic cells are critical steps for their respective success within cell‐based bone tissue engineering techniques 24. Previously, we found that Hst1 promoted the spreading of osteogenic cells on both bio‐inert substrates and titanium SLA surfaces *in vitro*. In this study, we found that the co‐administration of ATRA and Hst1 significantly increased cell spreading efficiency compared to the presence of Hst1 only.

In line with previous work, cell spreading was used as a key parameter to evaluate efficacy of surface compatibility for osteogenic cells by different bioactive agents *in vitro*. In our previous studies, we have used the percentage of spreading cells or cell index as parameters to evaluate cell spreading 14, 15. The former parameter indicates the percentage of cells that initiate protrusion, and the latter parameter qualifies the impedance of cells that proportionally correlate to, but not directly show, cell spreading extent. In contrast, in the current study, we adopted a point‐counting method 25 to directly measure the surface area of cell spreading parts, which could directly reflect the newly formed cell–substrate contact area. We further subtracted the area of nuclei to purely evaluate area of spreading part, which helped us to more precisely evaluate the spreading extent.

Most of the current methods to promote cell–substrate interaction are focused to modify the surface chemistry and/or topography 26. However, due to the large variety of biomaterials, there is still an apparent great need for broadly applicable approaches to promote cell spreading on biomaterials. Previously, we and others showed that Hst1 promoted adhesion, spreading, and migration of various epithelial cells from different origins, such as mucosa 12, 16, gingiva 14, 15, cornea 13, and skin 27, endothelial cells 14, 17, and osteogenic cells 11, 14. All together, these findings underline a non‐cell type‐specific character of Hst1 rendering a promising application potential for tissue engineering purposes. Next to Hst1, ATRA was used as agent to promote cell spreading for cell‐based tissue engineering techniques. Numerous studies have demonstrated that the RA signaling pathway, which is mediated via RAR and/or RXR, can modulate the expression of genes involved in cell growth, 28 energy metabolism, 24 and immune responses 29, 30. In a previous study, we found that a 3‐day treatment of ATRA caused uniformly‐directionally alignment of actin *in vitro* (Fig. S2). Notably, it was reported that ATRA increased the adhesion and spreading of pancreatic stellate cells via RARβ‐dependent signaling, thereby inhibiting cancer cell invasion 31. In this process, ATRA‐treated pancreatic stellate cells formed larger focal adhesion complexes, spread faster, attained a larger spreading area, attached stronger to the ECM (extracellular matrix), and displayed significantly larger and brighter focal adhesion complexes (both for talin and paxillin) in comparison with untreated control cells 31.

Here, we showed that RARs were potentially involved for the promoting effect of ATRA on the spreading of pre‐osteoblasts. For this purpose, we adopted specific antagonists of RARα, RARβ, and RARγ and found that the antagonists of RARα (ER‐50891) and RARβ (LE135), but not RARγ (MM‐11253), significantly suppressed cell spreading induced by co‐administered ATRA and Hst1. Furthermore, we found that the antagonists of RARα (ER‐50891) and RARβ (LE135) abolished the amplification by ATRA of Hst1's effects on cell spreading. These findings suggested that ATRA affects cell spreading by RARα‐ and RARβ‐dependent signaling. This may be consistent with the reports that the agonists of RARα and RARβ, but not RARγ, activated focal adhesion kinase (FAK) and paxillin in breast cancer cells 32.

Concerns may be raised for using ATRA since it was previously shown to have negative effects on the adhesion and migration of epithelial cells 33. Treatment of 0.1–1 μm ATRA for 1 h significantly inhibited the adhesion of retinal pigment epithelial cells. Furthermore, it was found that ATRA significantly inhibited the spreading of retinal pigment epithelial cells with suppressed FAK, suggesting that ATRA's effect is highly cell type‐dependent. Consequently, caution must be taken for extrapolating these data to osteogenic cells. Another concern may be that ATRA may inhibit the osteogenic activities of pre‐osteoblasts 34 and bone marrow stromal cells 35. In our previous studies, we showed that a long‐term (3–21 days) treatment with ATRA significantly reduced cell proliferation, metabolic activity, protein expression, osteocalcin expression, and extracellular matrix mineralization of osteogenic cells 20, 34, 35. In our current study, we showed that the short‐term (2 h) treatment of either Hst1 alone or ATRA alone did not result in significantly higher DNA content compared to the control. Surprisingly, the DNA content in the group of the co‐administered ATRA and Hst1 was significantly higher than those in the groups of ATRA alone, Hst1 alone, or control, which suggested the co‐administration of ATRA and Hst1 synergistically promoted cell attachment. Our data further showed that the 2‐h co‐administration of ATRA and Hst1 resulted in significantly enhanced metabolic activity of pre‐osteoblasts within the monitoring time span (5 days) than either ATRA or Hst1 alone. Moreover, neither ATRA alone nor Hst1 alone had any effect on ALP activity, suggesting that neither of them significantly influence osteogenic differentiation of pre‐osteoblasts. In contrast, the combination of ATRA and Hst1 significantly enhanced ALP activity. These data indicate that such a treatment with ATRA and Hst1 is potentially suitable to promote both the cell–substrate interactions of pre‐osteoblasts and enhance their osteogenic differentiation.

The underlying molecular mechanisms of ATRA and Hst1 co‐administration on ALP activity remain to be elucidated. Possibly, the activation of p38 MAPK signaling pathway may be involved. Recently, we found that specific p38 MAPK inhibitors abolish the promoting effect of Hst1 (data not shown) in this type of pre‐osteoblasts, suggesting that Hst1 could activate p38 signaling. It is well‐established that p38 MAPK is a key mediator for many drugs to upregulate ALP activity in pre‐osteoblasts 36, 37, 38. On the other hand, ATRA is also found to transiently activate p38 signaling 39, 40. Although a short‐term treatment of either Hst1 or ATRA seemed not sufficient to induce ALP activity, in combination Hst1 and ATRA significantly upregulated ALP (Fig. 5), suggesting an additive stimulating effect on p38 MAPK signaling. Further studies are needed to confirm this hypothesis. With the inspiration of the ALP result, we, thereafter, performed an experiment of extracellular matrix mineralization with osteogenic medium to check the effect of the 2‐h co‐administration of ATRA and hst1. We found that the 2‐h co‐administration of ATRA and hst1 was associated with a higher (without statistical difference) mineralization at 21 days post‐treatment than the control group (data not shown). In fact, the result is not so surprising since the effect of a 2‐h treatment can quickly taper during the 21‐day culture period with osteogenic medium (10% FBS‐containing α‐MEM with beta‐glycerophosphate and l‐ascorbic acid‐2‐phosphate as supplements). Consequently, the short‐term co‐administration of ATRA and hst1 can show a significant effect only in the initial cellular events of osteogenic activities, such as cell adhesion, spreading, proliferation, and early differentiation. For late (e.g., osteocalcin expression) and final differentiation (extracellular matrix mineralization), osteoinductive growth factors, such as bone morphogenetic proteins, are highly needed.

Finally, in this study we used a mouse MC3T3‐E1 cell line. Although MC3T3‐E1 cells are widely used as a cell model for pre‐osteoblasts, these effects must be replicated in systems that bear more relevance for the human physiological situation, such as primary mesenchymal stem cells and osteoblasts. Furthermore, caution should be taken to extrapolate the current *in vitro* results to *in vivo* situation. Animal studies are highly needed to confirm the promoting effect of co‐administered ATRA and Hst1.

In summary, our current study showed that Hst1 and ATRA co‐administration positively influenced the spreading, cellular metabolic activity, proliferation, and osteogenic differentiation of pre‐osteoblasts. Based on these observations, we postulated that such combined treatment may be supportive for cell‐based bone tissue engineering techniques.

## Conflict of interest

The authors declare no conflict of interest.

## Author contributions

GW and ECIV contributed to conceptualization; WS, GW, HL, AS, and DM participated in investigation; GW and JGMB collected resources; WS, AS, and GW performed formal analysis; KN and FJB curated the data; WS, HL, FJB, and GW wrote the original draft; AS, WS, DM, FJB, JGMB, HL, KN, and ECIV edited the manuscript; and WS, HL, GW, and ECIV acquired funding.

## Supporting information


**Fig. S1.** Fluorescent micrographs depicting the spreading of pre‐osteoblasts (stained with FITC‐Phalloidin) with or without a treatment with 1 µM ATRA for 3 days. Bar = 50μm.
**Fig. S2.** Graph depicting a point‐counting method to measure the surface area of cell spreading. The grid was randomly put on the light micrographs of cells during spreading for the point‐counting method. The filopodia and lamellipodia (red arrow) was included for calculating the cell spreading area with the exclusion of the relatively constant peri‐nuclear area (within red dot circle). Bar = 50μm.
