# Supplementary material for: All‐trans retinoic acid and human salivary histatin‐1 promote the spreading and osteogenic activities of pre‐osteoblasts in vitro
Source: FEBS Open Bio. 2020 Feb 11;10(3):396–406. doi: 10.1002/2211-5463.12792 (PMC7050254; doi:10.1002/2211-5463.12792)
Supplement: Supplementary file 1 — Fig. S1. Fluorescent micrographs depicting the spreading of pre‐osteoblasts (stained with FITC‐Phalloidin) with or without a treatment with 1 µM ATRA for 3 days. Bar = 50μm. Fig. S2. Graph depicting a point‐counting method to measure the surface area of cell spreading. The grid was randomly put on the light micrographs of cells during spreading for the point‐counting method. The filopodia and lamellipodia (red arrow) was included for calculating the cell spreading area with the exclusion of the relatively constant peri‐nuclear area (within red dot circle). Bar = 50μm. [file FEB4-10-396-s001.docx]

Appendix A


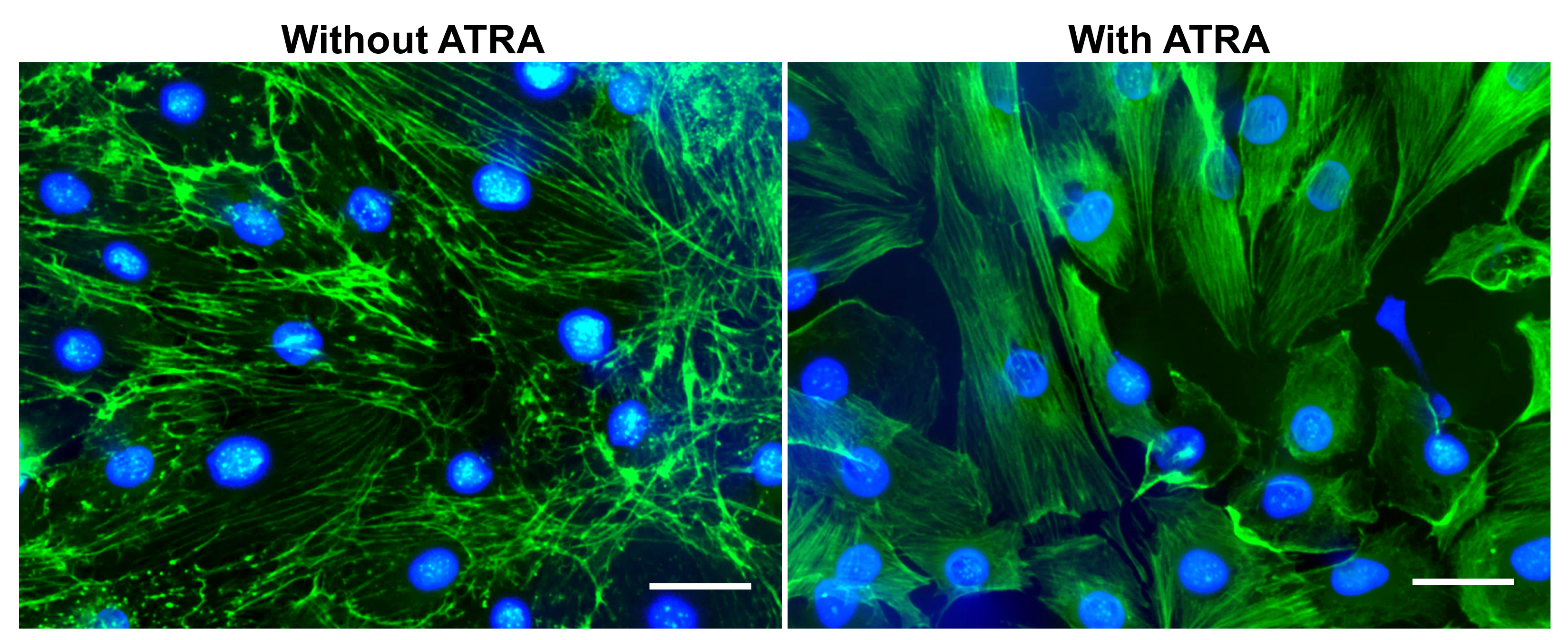


**Figure S1.** Fluorescent micrographs depicting the spreading of pre-osteoblasts (stained with FITC-Phalloidin) with or without a treatment with 1 µM ATRA for 3 days. Bar = 50μm

Appendix B

**Figure S2.** Graph depicting a point-counting method to measure the surface area of cell spreading. The grid was randomly put on the light micrographs of cells during spreading for the point-counting method. The filopodia and lamellipodia (red arrow) was included for calculating the cell spreading area with the exclusion of the relatively constant peri-nuclear area (within red dot circle). Bar = 50μm
